# Tuning the Performance of Metallic Auxetic Metamaterials by Using Buckling and Plasticity

**DOI:** 10.3390/ma9010054

**Published:** 2016-01-18

**Authors:** Arash Ghaedizadeh, Jianhu Shen, Xin Ren, Yi Min Xie

**Affiliations:** 1 Centre for Innovative Structures and Materials, School of Engineering, RMIT University, GPO Box 2476, Melbourne 3001, Australia; s3393723@student.rmit.edu.au (A.G.); jianhu.shen@rmit.edu.au (J.S.); s3460277@student.rmit.edu.au (X.R.); 2 Key Laboratory of Traffic Safety on Track, School of Traffic & Transportation Engineering, Central South University, Changsha 410075, China

**Keywords:** mechanical metamaterial, auxetic, buckling, large deformation, plasticity

## Abstract

Metallic auxetic metamaterials are of great potential to be used in many applications because of their superior mechanical performance to elastomer-based auxetic materials. Due to the limited knowledge on this new type of materials under large plastic deformation, the implementation of such materials in practical applications remains elusive. In contrast to the elastomer-based metamaterials, metallic ones possess new features as a result of the nonlinear deformation of their metallic microstructures under large deformation. The loss of auxetic behavior in metallic metamaterials led us to carry out a numerical and experimental study to investigate the mechanism of the observed phenomenon. A general approach was proposed to tune the performance of auxetic metallic metamaterials undergoing large plastic deformation using buckling behavior and the plasticity of base material. Both experiments and finite element simulations were used to verify the effectiveness of the developed approach. By employing this approach, a 2D auxetic metamaterial was derived from a regular square lattice. Then, by altering the initial geometry of microstructure with the desired buckling pattern, the metallic metamaterials exhibit auxetic behavior with tuneable mechanical properties. A systematic parametric study using the validated finite element models was conducted to reveal the novel features of metallic auxetic metamaterials undergoing large plastic deformation. The results of this study provide a useful guideline for the design of 2D metallic auxetic metamaterials for various applications.

## 1. Introduction

Four fundamental mechanical properties of materials in isotropic elasticity are Poisson’s ratio (ν), Young’s modulus (E), shear modulus (G), and bulk modulus (K). Note that these four parameters are interrelated, and the applicability of the theory of elasticity is limited to stress-strain conditions wherein the stress is below the yield point. Young’s modulus is the measure of stiffness in the linear elastic range. Poisson’s ratio is the least studied among these elastic constants. However, it governs the deformation feature of materials under various loading conditions [1,2]. This property is represented by the negative of the ratio between transverse and longitudinal strains [3]. The majorities of materials have a positive Poisson’s ratio that is about 0.5 for rubber and 0.3 for glass and steel [3]. The thermodynamic requirement in the theory of elasticity for a conservative system demonstrates that for homogeneous isotropic materials, the theoretical bound of Poisson’s ratio is from −1 to 0.5. Therefore, the existence of materials with negative Poisson’ ratio (NPR) has long been accepted and they are known as “auxetic” materials [3,4]. The deformation feature of auxetic materials can be described as an anomalous behavior that indicates that materials expand (contract) in a transverse direction when uniaxially stretched (compressed) [3,4,5,6,7]. Based on their origin, auxetic materials can be classified as naturally occurring or artificial. The exotic mechanical properties of the natural auxetic materials inspired plenty of researchers to search for the underlying mechanisms that cause auxetic behavior. Those findings were applied to the design of artificial materials [1,8].

Many cellular structures were designed to have exceptional properties. When these properties are superior to these found in nature, the artificial cellular material is termed as a metamaterial. Metamaterials gain their uncommon and unique properties from geometrical configurations rather than chemical composition [9,10]. Furthermore, the behavior of metamaterials was normally explained in terms of the intricate interplay between their microstructures and their deformation mechanisms [11,12].

Over the past decades, continuous efforts were put in towards the development of auxetic metamaterials because of their unconventional behavior under uniaxial loading. Their unique properties opened a window towards a wide range of applications such as the design of novel fasteners [13], biomedical applications [14], energy-absorbing devices [15], acoustic dampers [16], membrane filters with variable permeability [17], and the design of composites [18].

Macroscopic auxetic cellular structures with 2D re-entrant honeycombs cells were firstly presented by Gibson and Ashby [19]. Then an artificially designed auxetic foam with a re-entrant cell was reported in the seminal research of Lakes in 1987 [3]. Following that, the interest of researchers was concentrated on periodic 2D auxetic materials through modifying the geometry of microstructures. These consist of composites with star-shaped inclusions [20], a structure formed from lozenge grids [21], a formed structure from square grids [22], square lattice of circular holes [11], and many other 2D topological patterns. Due to the notable challenge in the fabrication of 3D auxetic materials that consist of a microstructure with complicated geometries, only a small number of synthetic 3D auxetic metamaterials have been fabricated [10]. Recently an auxetic cube using “buckliball” as the building cell by Babaee *et al*. [23] and auxetic cube with simple spherical by Shen *et al*. [10] were invented, manufactured, and tested. It is worth noting that, most of these 2D and 3D auxetic metamaterials were constructed with soft elastic base materials. They deformed elastically under uniaxial loading and the deformation was reversible and repeatable. However, the weak stiffness and strength and the low softening temperature of these elastomeric metamaterials prevented them from many applications requiring high stress and high temperature. In contrast to elastomers, metals have higher stiffness, strength, density, and melting point. Copper, gold, platinum, silver, and brass are especially ductile and might be suitable for fabricating novel auxetic metamaterials. There is a great potential for metallic auxetic metamaterials to be used in engineering products.

Only a few researchers studied the auxetic performance of metallic auxetic metamaterials undergoing large plastic deformation. Therefore, knowledge of the deformation features and auxetic performance of metallic metamaterials is very limited. The first work in this area was done by Friis *et al*. [5], who proposed a polymeric and metallic foam with auxetic behavior. Recently, a 2D metallic auxetic periodic structure with low porosity was reported by Taylor *et al*. [24]. Another study in this area was carried out by Dirrenberger *et al*. [25] on the auxetic behavior of the metamaterials undergoing plastic deformation by using an anisotropic compressible plasticity framework as a macroscopic model. Moreover, the behavior of elastoplastic auxetic microstructural arrays was assessed employing a continuum-based micromechanical model by Gilat *et al*. [26].

Inspired by previous works on buckling-induced auxetic elastic and elastoplastic metamaterials [10,11,23,27,28,29,30], we expanded the similar design approach to metallic structures. However, the buckling-induced geometrical design lost its auxetic behavior under large plastic deformation when the base material was changed to metals. Therefore, the specific aim of this research is to develop a general approach to designing and tuning the metallic metamaterials with auxetic performance undergoing large plastic deformation. The auxetic performance of metamaterials on any length of scale is dependent on the geometrical features and deformation mechanisms of their microstructures. Hence, the general approach of the metamaterial starts at the microstructure level by employing an existing deformation mechanism that leads to auxetic behavior [10,11]. In this approach, the geometry of microstructure of a regular structure is modified then it is altered by the desired buckling modes. A new category of 2D metallic auxetic metamaterials over a large strain range can be obtained by using different scale factors. The new metallic metamaterial exhibits auxetic behavior with almost constant negative values of Poisson’s ratio over a large strain range. This feature is very useful for practical applications in macroscopic metamaterials as well as in nanoscale structures [31,32]. A set of parametric studies using validated finite element (FE) simulation have been done to show the possibility of providing an effective control method to tune the mechanical properties of the 2D metallic auxetic metamaterial during the designing process.

## 2. A General Approach to Tuning the Performance of 2D Metallic Auxetic Metamaterial

In order to obtain a novel class of 2D metallic auxetic metamaterials with the potential to retain tuneable auxetic behavior under a wide range of applied strains, a general systematic approach was developed based on our previous work [10]. This general approach leads to the possibility of tuning the auxetic behavior and mechanical properties for the targeted applications. The key idea of this methodology is the exploitation of a pattern from the linear buckling analysis to create microstructures of metamaterials with an auxetic response. This methodology can be divided into four steps as described in the following sections. Moreover, the magnitude of alteration of microstructure and plastic properties of the base material were studied as the primary key factors in tuning the performance of 2D metallic auxetic metamaterial. These results are presented in the parametric study section.

### 2.1. Initial Geometric Design of Microstructures Modified from a Regular Pattern

The auxetic performance of a metamaterial was determined by the geometrical features and deformation mechanisms of its microstructure. According to the geometry of microstructure of metamaterials that exhibited auxetic behavior induced by elastic instability, the ribs of their building cell were slightly thicker in the proximity of their connecting points rather than the middle parts of the ribs [10,11,23,27]. Consequently, rotation occurred at the middle parts of these ribs in clockwise and counter-clockwise directions, to form a deformation mechanism after the buckling of the ribs [33,34]. These observations and mechanisms led us to think that buckling-induced auxetic cellular metamaterials could be produced from a conventional regular cellular lattice through geometrical modification of their microstructures.

**Figure 1:**
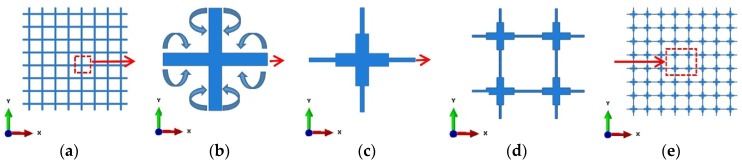
(**a**) Conventional regular lattice; (**b**) building unit cell of initial regular lattice; (**c**) modified unit building cell of microstructure; (**d**) representative volume element; (**e**) designed bulk metamaterial with cross-shaped microstructures for experiments.

In this research the microstructure of a new buckling-induced 2D auxetic metamaterial was produced from an existing conventional regular lattice through the proposed modification. This method consists of moving a small portion of the middle part of their ribs to the proximity of the connecting joints. This geometrical modification on the microstructure was based on the hypothesis that, under deformation, joints behaved as “rigid joints” and rotated relatively to each other after the ribs buckled. The geometric modification of the unit cell of the regular lattice through the proposed method is illustrated in Figure 1b,c. The new buckling-induced 2D auxetic metamaterial was similar to the buckling-induced auxetic metamaterials investigated by Bertoldi *et al*. [11]. According to the buckling pattern, the representative volume element (RVE) contained four unit cells as shown in Figure 1d. Similar to the designing approach of elastic metamaterials [10], the bulk 2D metamaterial was constructed by replicating RVEs along two planar directions, as shown in Figure 1e.

### 2.2. Investigation of the Instability of Initial 2D Auxetic Metamaterial Using a Linear Perturbation Procedure (Linear Buckling Analysis)

The elastic instability in elastomeric metamaterials triggered a pattern switching phenomenon above a critical load, which led to auxetic behavior under compression [28]. In order to identify the desired buckling mode, finite element analysis (FEA) was performed on the existing cross lattice and the modified periodic cellular metamaterial. The stability of these microstructures was evaluated using commercial finite element software ABAQUS with standard solver (Simulia, Providence, RI, USA) and a linear perturbation procedure was conducted to find the critical loads (eigenvalues) for different buckling modes (Eigen modes). The numerical models were constructed with linear solid elements of the secondary accuracy (element C3D8 with a mesh sweeping seed size of 0.4 mm). It should be noted that the applied boundary conditions influence the buckling modes of the finite sized numerical model. In this study, all degrees of freedom on the top and bottom nodes of the numerical model were constrained except for the nodal movement of the top surface in the direction of the applied load. The analysis was carried out to simulate the standard uniaxial compression. It is worth noting that for purely elastic models the buckling modes were independent of the modulus of elasticity; thus any value of modulus could be used to conduct the buckling analysis. Therefore, the proposed design approach was independent of the influences of the elastic base material. The Young’s modulus of elasticity and Poisson’s ratio of brass were used as the properties of the base material in numerical simulations.

### 2.3. Identification of the Desired Buckling Pattern from an Elastic Instability Analysis

As mentioned before, the linear buckling analysis yields a number of eigenvalues and buckling mode eigenvectors corresponding to these eigenvalues. Normally the lowest eigenvalue with the corresponding buckling mode is of interest because the buckling modes with higher eigenvalues are difficult to trigger under quasi-static loading. The desired buckling modes, termed alternating periodic buckling mode, were identified through the deformation features of buckling-induced auxetic metamaterials previously investigated [10,11,23,35]. In most cases, the auxetic behavior of this type of metamaterials was featured by such a mode with alternating mutually orthogonal ellipses. According to this identified principle, the seventh mode shape of initial metamaterial (regular conventional lattice) is similar to the observed buckling pattern that leads to the auxetic behavior. In contrast, the first buckling mode shape of the newly designed metamaterial (with the lowest eigenvalue) can be adopted as the desired buckling mode shape.

### 2.4. Design New Metamaterials through Superposition of Desired Buckling Mode

**Figure 2:**
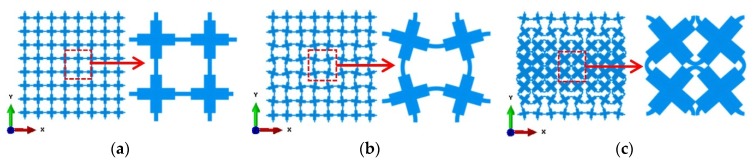
Definition of PSF to alter the initial microstructure of a buckling-induced metamaterial. The normalized desired buckling mode shapes scaled by different corresponding DSFs and their most central RVEs. (**a**) PSF = 0, DSF = 0; (**b**) PSF = 20%, DSF = 0.00184; (**c**) PSF = 100%, DSF = 0.0092.

Previous research on designing elastic buckling-induced auxetic metamaterials revealed that altering the initial geometry of the microstructure was a way to trigger the deformation to a specific pattern that led to auxetic behavior [10,34]. One set of the output of the buckling analysis was a number of non-dimensional buckling modes. Thus, in order to define the magnitude of alteration of microstructure, a scale factor is required. In this study, the pattern scale factor (PSF) was proposed to add the desired buckling mode shape to the initial geometry of the metamaterial. To quantify the magnitude of the alternation, the PSF was determined using the special geometric feature of an RVE of the microstructure initially designed. The innermost RVE of the bulk metamaterial was selected to quantify the PSF. The final microstructures for metallic auxetic metamaterials were obtained by adding the desired mode shape multiplied by the specified design scale factor (DSF) to the original coordinates. DSF is an independent factor that consists of scaling the maximum displacement amplitude to visualize a deformed shape. When the walls at the center of the RVE touched each other, the corresponding DSF (in this case 0.0092) was defined as PSF = 100%, as shown in Figure 2c. Other magnitudes of PSF were defined accordingly as 0% and 20%, corresponding to DSFs of 0 and 0.00184, respectively. The normalized desired buckling mode and its innermost RVE with different PSFs are presented in Figure 2. Through changing the PSF, a new class of auxetic metamaterials with different auxetic performance was produced [10]. The tuneable auxetic performance was a special feature of this class of auxetic metamaterials and potentially could be implemented in particular applications with a specific negative value of Poisson’s ratio. It should be noted that, without this alternation, the deformation of this type of auxetic metamaterial was uniform at the beginning stage under compression, which exhibited a positive Poisson’s ratio until buckling occurred [10]. In our previous work [10], the desired buckling mode of the bulk metamaterial was superposed to the original designed microstructures of metamaterial. It resulted in a non-periodic microstructure. The periodicity was achieved by patterning the altered central RVE of the bulk metamaterial in this study. The altered bulk metamaterial was formed by repeating this altered RVE as the unit cell. 

## 3. Experiments

### 3.1. Fabrication of 2D Metamaterials for Experiments

**Figure 3:**
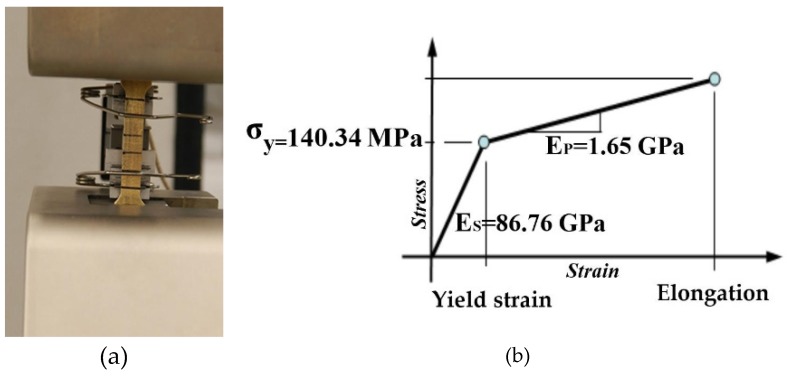
Tensile test of 3D printed brass dog-bone using MTS machine. (**a**) Front view of brass dog-bone. (**b**) Schematic curve of stress-strain for brass.

The metallic specimens were fabricated using a high-resolution 3D printing technique. In the first step, the metamaterial was printed in wax then the wax model was put inside liquid plaster. After setting the plaster, it was placed in an industrial oven to melt out the wax. The wax was used as a supporting material to make a plaster mold. Eventually, the molten metal was poured into the plaster mold and was placed at the fixed position to become solid. Raw brass was selected as a representative base material of printed specimens because of its excellent ductility among available materials for 3D printing. The mechanical properties of brass were obtained directly from a standard tensile test on six dog-bone specimens using an MTS (material test system) machine (MTS Company, Eden Prairie, MN, USA), which is shown in Figure 3. The average mechanical properties of brass tests are summarized in Table 1. The influence of the potential anisotropy of base material is not studied here. This potential may be caused by the inevitable inclusion of air bubbles into brass specimens during the manufacturing process. The penetration of tiny bubbles causes very small holes inside the specimens and changes the mechanical properties and density of different printed specimens. However, the excellent agreements between the numerical and experimental results revealed that the influence of potential anisotropy is negligible in this study.

**Table 1:** Material properties of brass.

Modulus of Elasticity (GPa)	Yield Stress (MPa)	Strain Hardening Modulus (GPa)	Density (GPa)
86.76	140.34	1.65	8720

**Figure 4:**
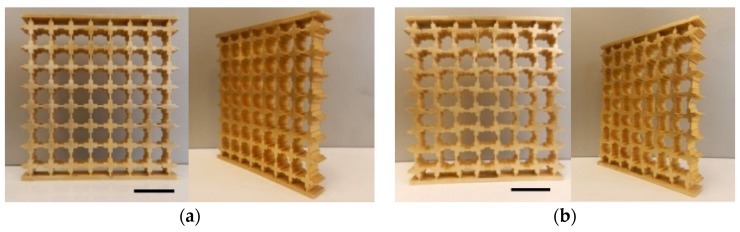

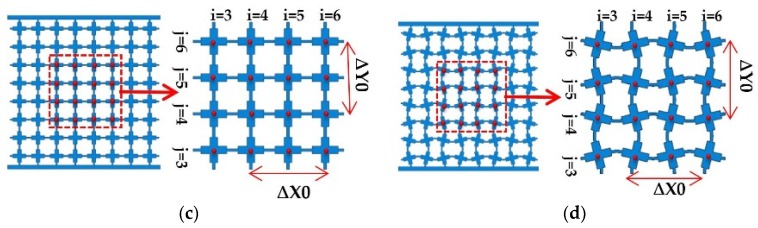
The final design of metamaterial employed for FEA and experimental investigation. (**a**) Front view and perspective view of buckling-induced metamaterial (PSF = 0%; density: 2798.05 kg m^−3^, overall mass: 228.5 g, mass error: 1.08%, height × width × depth: 92.8 mm × 88 mm × 10 mm, Scale bar: 20 mm); (**b**) front view and perspective view of the metamaterial with altered geometry (PSF = 20%, overall mass: 222.57 g, density: 2725.43 kg m^−3^, mass error: 2.06%, height × width × depth: 92.8 mm × 88 mm × 10 mm, Scale bar: 20 mm); (**c**) schematic diagram of central region with 16 nodes (PSF = 0%); (**d**) schematic diagram of central region with 16 nodes (PSF = 20%). The image processing was used to measure the horizontal and vertical center-to-center distances of nodes.

**Figure 5:**
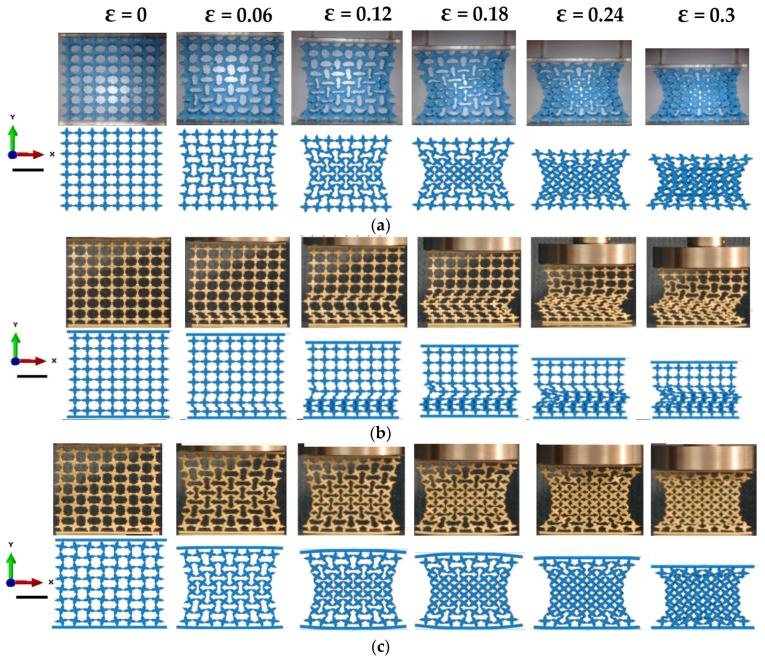
Comparison of deformation patterns of three new designed metamaterials between numerical and experimental results. (**a**) Experimental and numerical results of initial design of auxetic elastic metamaterial (rubber) with PSF = 0%; (**b**) experimental and numerical results of initial design of metallic metamaterial with PSF = 0%; (**c**) experimental and numerical results of the new design of auxetic metallic metamaterial with PSF = 20% (Scale bar: 20 mm, load direction Y, strain rate: 5 × 10*^−^*^3^ S*^−^*^1^).

The 2D periodic bulk metamaterials for original buckling-induced design (PSF of 0%) and for an altered one with unit cells with a PSF of 20% are shown in Figure 4.The first 3D printed brass sample is a buckling-induced metamaterial with PSF = 0, as shown in Figure 4a, and the other is the altered one that was superposed by the desired buckling mode with a PSF of 20%, is shown in Figure 4b. The dimensions of the first and second test specimens were height × width × depth = 92.8 mm × 88 mm × 10 mm. Two plates were added to the bulk materials to constrain the degrees of freedom of the top and bottom nodes’ surfaces except for a degree of freedom in the direction of load. To check the accuracy of the manufactured samples, the weights of printed models were measured and compared with the weight of our initial design obtained from the numerical mode. The weights of printed samples were slightly heavier than the designed ones. A numerical comparison of models with the different weight obtained by changing the void of RVE confirmed that these small weight differences were negligible. In order to compare the deformation patterns of rubber and metallic metamaterials with initial geometric design (PSF = 0%), a 2D elastomeric specimen was manufactured using silicon-based rubber (Tango plus, In’Tech, Ramsey, MN, USA), as shown in the top-most row of Figure 5. The material properties of silicon-based rubber (Tango plus) were measured in reference [10].

### 3.2. Experimental Setup for Uniaxial Compression Testing of 2D Metallic Metamaterials

Quasi-static uniaxial compression tests were carried out to investigate the performance of 2D metallic auxetic metamaterials under compression. The tensile tests were conducted by a Shimadzu machine (Shimadzu Company, Kyoto, Japan) at a fixed strain rate of 5 × 10^−3^ s^−1^. The deformation process was captured by two cameras. The first camera was used to capture photos in the lateral direction of our 2D test specimens every 30 s. The second camera was employed to record a performance video of the test process. The nonlinear response of metamaterials was considered to be in the range of effective auxetic strain. In other words, the focus of this research was on the behavior of metamaterials, whereas the value of nominal applied strain was below the densification strain. Densification strain was defined as the maximum value of applied strain that satisfies the condition of maximum energy efficiency, as used by Shen *et al*. [36]. The experimental value of Poisson’s ratio was calculated through image processing from the nodes in the central parts of ligaments, as shown in Figure 4c,d. Indeed, at a specific value of nominal strain, the $\left(x_{i,j},\;y_{i,j}\right)$ coordinates of the centroid point of each unit cell were determined. The row and column indices are represented by 1≪i≪**8** and 1≪j≪**8**, respectively. The central area under consideration consists of 16 nodes; these are designated with a red dashed line indicating 3≪i≪ 6 and 3≪j≪6, as shown in Figure 4c,d. The horizontal and vertical centroid-to-centroid distances were calculated by using coordinates$\;\left(x_{i,j},y_{i,j}\right)$, $\Delta x_{i,j}=x_{i+1,j}-x_{i-1,j}$ and $\Delta y_{i,j}=y_{i,j+1}-y_{i,j-1}$. Also, the center to center distances between the points of undeformed unit cells before compression were defined by Δx(0)=Δy(0)=11 mm. Equation (1) was employed to calculate the local values of the engineering strain Poisson’s ratio.

(1) $$\upsilon_{i,j}=-\frac{\frac{\Delta X_{i,j}}{\Delta x\left(0\right)}}{\frac{\Delta Y_{i,j}}{\Delta y\left(0\right)}}$$

Eventually, four values of Poisson’s ratio were calculated from the central nodes under consideration, and the average of them was computed at each specific value of nominal strain. The deformation pattern of elastic buckling-induced auxetic metamaterial with a rubber base material is shown in Figure 5a, which is similar to the pattern observed by Bertoldi *et al.* [11].

### 3.3. Experimental Results

**Figure 6:**
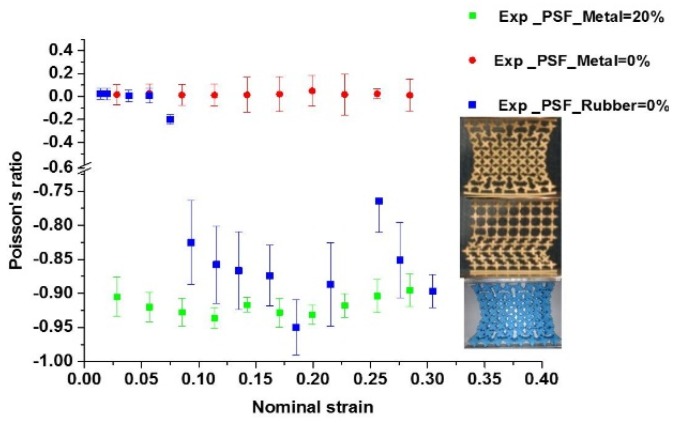
Comparison of auxetic and non-auxetic behavior of new design of auxetic metallic metamaterial with PSF = 20%, initial design of metallic metamaterial with PSF = 0 and initial design of auxetic elastic metamaterial (rubber) with PSF = 0% (Scale bar: 20 mm, load direction Y, strain rate: 5 × 10*^−^*^3^ S*^−^*^1^).

As expected, the deformation pattern of a brass metamaterial undergoing plastic flow was different from that of a rubber sample with only elastic deformation. When the base material was changed to metal, the auxetic behavior disappeared, and a new non-auxetic pattern similar to the global buckling pattern for elastic bulk materials [10] was observed, as shown in Figure 5b. This pattern usually yielded a zero or positive Poisson’s ratio. In contrast, the test specimen with altered microstructures (PSF = 20%) exhibited obvious auxetic behavior, as shown in Figure 5c. It developed from a localized buckling pattern and was similar to mutual ellipses with long orthogonal axes. The results for all samples are presented in Figure 6 as a function of nominal strain. These experimental results revealed the significant influence of base materials with nonlinear properties or metal plasticity on the auxetic behavior of the buckling-induced auxetic materials. They also proved the effectiveness of designing the metallic auxetic metamaterials using a buckling mode to alter the initial geometry of buckling-induced metamaterials. By applying different values of PSF, a family of metallic metamaterials were generated, which exhibited obvious auxetic behavior nearly in the full range of its deformation process, as illustrated in Figure 6. A detailed analysis on the deformation process of the metallic specimen was conducted to reveal the reason behind the loss of auxetic behavior in Figure 5b. At the beginning of the compression process (*ε* < 0.06), the initial localization of plastic deformation occurred within the bottom layer of cells, and they were crushed firstly. As compression progressed, more localization took place within the central layers until all layers of the specimen were crushed and a non-auxetic deformation pattern was formed, as shown in Figure 5b. The observed crushing process also showed that the localized plastic deformation did not propagate layer by layer. This deformation localization triggered a different pattern without auxetic behavior. Thus, the key feature of the observed pattern in the brass specimen with PSF = 0% was the localization of plastic deformation. Inherently, deformation localization in one layer was caused by the sudden decrease of loading modulus of the metallic base material. The starting layer for this localization was determined by the manufacture imperfection in the brass specimen.

## 4. FE Simulations

### 4.1. Post-Buckling Analysis and Validation of Numerical Models

FE simulations were conducted to further clarify the loss of auxetic behavior of metallic buckling-induced metamaterials and to reveal the features observed in experiments on the new metallic auxetic metamaterials. A nonlinear post-buckling analysis on all of those metamaterials under uniaxial compression was carried out using ABAQUS/Explicit (Simulia, Providence, RI, USA). It should be noted that the results of post-buckling analyses were affected by the effects of complex self-contact and large deformations. Remarkably, the complex self-contact was an unavoidable factor for large deformation analysis [37]. To overcome this obstacle, the explicit simulation was carried out with a prescribed velocity profile. The mechanical properties of brass were set as a bilinear material model in FEA. In numerical models, the classical metal plasticity model was used to define the yield, hardening rule, and inelastic flow of the metamaterials at relatively low temperatures. The plasticity behavior of this model was simplified as a bilinear isotropic hardening behavior. The von Mises yield surface is used to measure isotropic yielding by defining the exact value of yield stress as a function of uniaxial equivalent plastic strain. The inertia effect was minimized by defining amplitude to apply the velocity on top of the FE model as it was used in reference [37]. The velocity was applied gradually and the acceleration at the beginning and ending of compression process was zero [10].

At the first stage of post-buckling analysis, the FE simulations were carried out utilizing 3D solid elements, the same as the previous buckling analysis. The deformation process of the newly designed metallic auxetic metamaterial with PSF = 20% is shown in Figure 5c. A comparison of the deformation process between the experiments and the numerical model revealed an excellent agreement between them, as shown in Figure 5.

**Figure 7:**
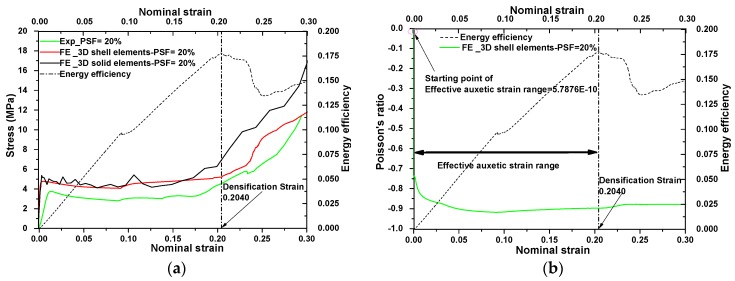
(**a**) Comparison of nominal stress-strain curves of the auxetic metamaterial between experiment and FE results (FE models with 3D shell elements and 3D solid elements) and using energy efficiency method to find densification strain; (**b**) defining the lower bound and upper bound of effective auxetic strain range.

Three-dimensional shell elements were used to reduce the computational costs. Four nodes—standard, linear square element with second order accuracy (S4R)—were used with at least five integration points at the minimum links in the FE model. The FE simulation results with shell elements were validated by comparing with experimental results and also with the result of previous post-buckling analyses with 3D solid elements, as shown in Figure 7a. They agreed with each other in general trend and features. However, the level of stress corresponding to FE results was higher than the stress level from the experimental results. These differences were attributed to the imperfection of the printed specimen in our experiments, which was supported by the large variation of properties, listed in our previous work [29].

### 4.2. Defining a Strain Range for Auxetic Metamaterials Using the Energy Efficiency Method

In this study, the effective auxetic strain range was defined as the strain between the starting point for negative value of Poisson’s ratio shown in Figure 7b and the densification point from stress-strain shown in Figure 7a,b. The starting point of effective auxetic strain range was defined as the first point that Poisson’s ratio began to decline as shown in Figure 7b.

The auxetic behavior of the metallic metamaterial started after application of a very small strain in the compression test, and this value was close to zero. The endpoint of the effective auxetic range is densification strain; this physically corresponds to the start point, from which a sharply rise of stress is observed in the stress-strain relationship as shown in Figure 7a. It is shown that from the densification strain onwards, the stress level in stress-strain curve rises sharply. The term “effective” is used to indicate two important features of the metallic auxetic metamaterials within the described strain range for their potential applications. One is that the nominal Poisson’s ratio is negative and similar, and the other is that the stress level over the entire deformation range is similar and below the densification strain. A relative constant plateau stress and similar Poisson’s ratio over the entire deformation range from the FE results was calculated using an energy efficiency method, as was used in references [36,38]. In this method, firstly the absorbed energy is defined as the total area under the stress-strain curve. Then, the energy efficiency parameter is found by dividing the absorbed energy by the stress itself, as shown below:

(2) $$E\left(\varepsilon \right)=\frac{\int_{0}^{\varepsilon }\sigma \left(\varepsilon \right)d_{\varepsilon }}{\sigma }$$

When the *E*(*ε*) reaches the maximum point, the corresponding strain is the densification strain. The densification strain is marked by a dashed line in Figure 7a,b. As a special feature, the fact that the densification strains from the experimental data are similar to those from the FE results also proved the accuracy of the FE models.

### 4.3. Parametric Studies

After the numerical models were validated, parametric studies were carried out to confirm the cause of loss of auxetic behavior for buckling-induced metallic specimens identified in the experiments. As for the pattern-altered specimen, the dominant factors to control the performance of metallic auxetic metamaterials were identified and qualified using the validated FE models. Based on experimental observations, the reason for the loss of auxetic behavior for metallic specimens was related to the plastic deformation of the buckling-induced auxetic metamaterials. The localization of the plastic deformation caused by buckling at an RVE length scale resulted in a global deformation mode rather than the desired buckling pattern in all RVEs. It was difficult to verify this assumption directly. However, two methods to prevent the localization of plastic strain could be applied to buckling-induced design. The first method is eliminating buckling by introducing a large imperfection; the second method is the increase of strain hardening modulus of its base material close to the elastic modulus. If those methods should be effective, the assumption would be proved indirectly. As mentioned before, the buckling mode alternation of the microstructure could be used as an innovative approach to design new metallic auxetic metamaterials. To enhance and facilitate its potential applications, an individual tuning method should be provided to assist with the design of a metallic metamaterial with a prescribed performance. Based on the feature of metamaterials, the change of Poisson’s ratio can be achieved by adjusting the geometrical configuration of microstructures, while other mechanical properties can be achieved by changing the elastoplastic properties of its base material. It is difficult to individually change those features by experimental methods, so the validated FE model was used to do the parametric study.

**Figure 8:**
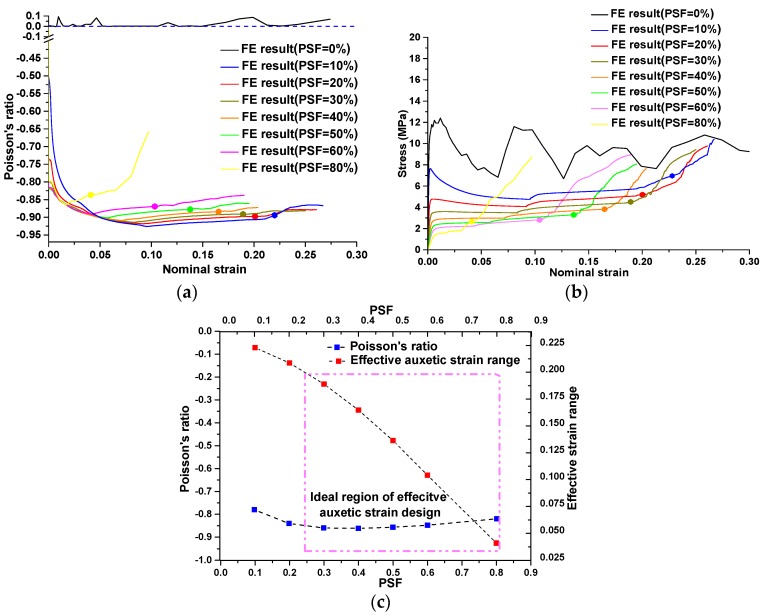
Parametric study on influence of PSF on auxetic behavior. (**a**) Evaluations of Poisson’s ratio as function of applied strain corresponding to different PSF; (**b**) stress-strain curves corresponding to different PSF; (**c**) average values of Poisson’s ratio and effective auxetic strain ranges corresponding to different PSF.

In this study, the magnitude of alteration was represented by PSF. As mentioned before, the PSF was a key parameter to alter the initial topology of RVE with desired buckling mode. The range of variation of PSF was between 0% and 100%. A series of systematic simulations were conducted to study the influence of PSF on auxetic performance of the new metallic auxetic metamaterials. The elastoplastic properties of brass were defined identically to the material properties in the experiment. The results are presented in Figure 8, in which the effective auxetic strain range is marked with round dots.

According to these results, it was determined that the increase of PSF resulted in the decrease of the densification strain and thus the effective auxetic strain range for auxetic behavior. This phenomenon was obvious, as shown in Figure 8c. By increasing the PSF, the mutual ellipses in RVE became smaller, thus the internal walls of the elliptical parts touched each other at a smaller strain. Therefore, the densification strains decreased accordingly.

The nonlinear response of Poisson’s ratio with respect to compressive strain is shown in Figure 8a with different PSF values. The negative values of Poisson’s ratio did not change much with respect to PSF, especially when the PSF are in the range of 10% to 60%. This finding revealed that the value of Poisson’s ratio is dominated by the deformation patterns initiated by the buckling mode applied. In this PSF range, the effective auxetic strain range can be individually designed with a prescribed negative value of Poisson’s ratio required by the aimed application, as illustrated in Figure 8c. An ideal region for individually altering the effective auxetic strain is defined and illustrated with a dashed rectangle in Figure 8c. Within this region, Poisson’s ratio maintained a constant negative value and the effective auxetic strain can be individually changed by the value of PSF.

**Figure 9:**
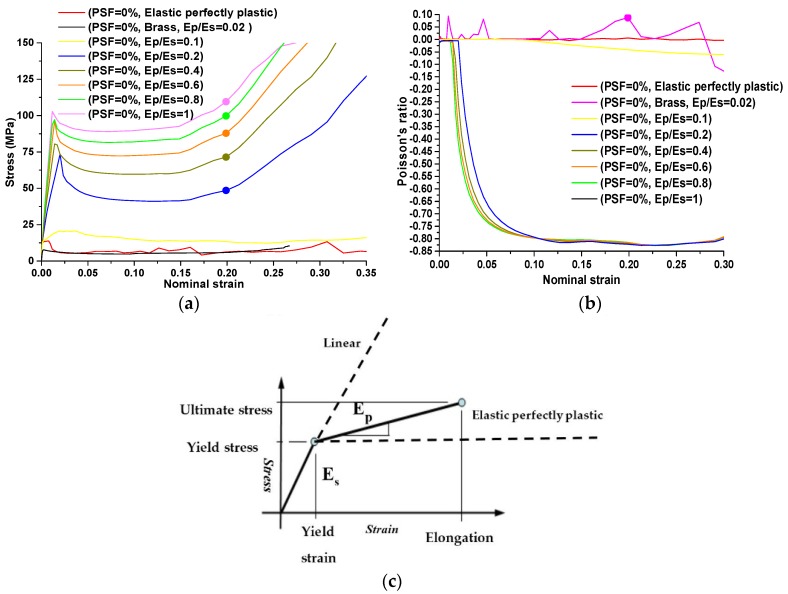
The results of a parametric study on the influence of plastic hardening on the recovery of auxetic behavior. (**a**) Evaluations of Poisson’s ratio as a function of applied strain corresponding to different hardening ratios for metamaterials with PSF = 0%.; (**b**) stress-strain curves corresponding to different hardening ratios for metamaterials with PSF = 0%; (**c**) schematic curve of stress-strain for metallic materials (strain hardening ratio).

As mentioned previously, the strain hardening of the base material could be used to prevent the localization of the plastic deformation caused by buckling at RVEs. Plastic strain hardening is a common phenomenon for metallic materials, which can be simplified as a linear relationship with respect to plastic strain. It is represented by the slope of this line, termed the strain hardening modulus (Ep), in the stress-strain curves shown in Figure 9c. In most cases, the stress-strain curve of a metallic material is determined by the elastic region (Es) as well as the plastic region (Ep). In this study, a simple ratio was defined as Ep/Es to characterize the effect of plastic hardening on the auxetic performance of our designed metamaterials. This ratio was termed the “plastic hardening ratio.” The variation range of this ratio is between 0 and 1.

**Figure 10:**
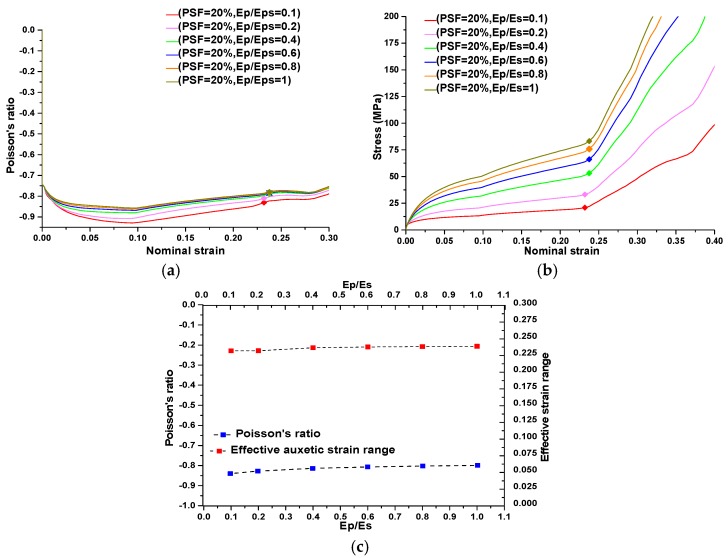
The results of a parametric study on the influence of plastic hardening on the auxetic behavior of a metamaterial with PSF = 20%. (**a**) Evaluations of Poisson’s ratio as a function of applied strain corresponding to different hardening ratios; (**b**) stress-strain curves corresponding to different hardening ratios; (**c**) average values of Poisson‘s ratio and effective auxetic strain ranges corresponding to different strain hardening ratios for metamaterials with PSF = 20%.

**Figure 11:**
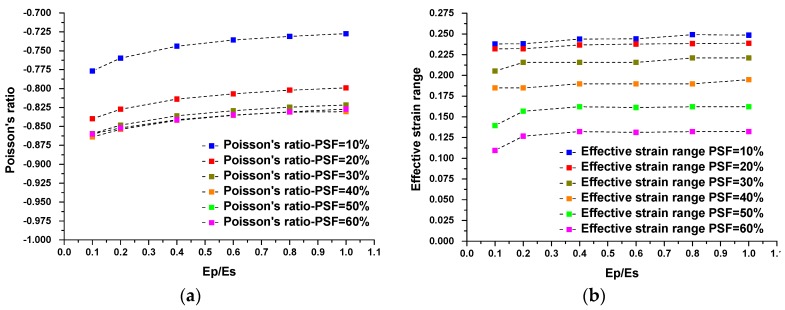
The results of a parametric study on the influence of pattern scale factors on auxetic behavior and effective auxetic strain range. (**a**) Evaluations of Poisson’s ratio as the function of hardening ratio corresponding to pattern scale factors; (**b**) evaluations of effective auxetic strain range as the function of hardening ratio corresponding to different pattern scale factors.

A ratio of 0 represents perfectly plastic material and a ratio of 1 represents purely elastic material. The result for a plastic hardening ratio of 1 with buckling-induced metamaterials confirmed that increasing the plastic hardening ratio will restore the auxetic behavior of buckling-induced metamaterials. As shown in Figure 5a, the experimental rubber specimen with PSF = 0% exhibited auxetic behavior under compression as a purely elastic material or when Ep/Es = 1. This finding was further validated by a numerical investigation on the initial geometry of buckling-induced metamaterial with PSF = 0%. The FE results for different values of Ep/Es at PSF = 0 are presented in Figure 9a,b. It is shown that the response of a metamaterial changed from non-auxetic to auxetic by increasing the plastic hardening ratio. Based on this evidence, the reason behind the loss of auxetic behavior was attributed to the localization of the plastic deformation caused by buckling at RVEs. To evaluate the effect of plasticity on the auxetic behavior of our newly designed metallic auxetic metamaterials, the hardening ratio was varied in the range of 0 to 1. The results of this numerical parametric study for the newly designed buckling-induced metamaterial with PSF = 20% are presented in Figure 10 and for other values of PSF are shown in Figure 11. Also, it should be mentioned that when Ep/Es tends to zero, the response is closer to the rotation of rigid squares and so the Poisson’s ratio tends to be −1 [39]. According to the numerical results shown in Figure 10 and Figure 11, it can be concluded that the difference of the calculated negative value of Poisson’s ratio for models with different strain hardening ratios were negligible. Also, the effective auxetic strain ranges for models with different hardening ratios were similar and the difference among these ranges can be neglected. However, the stress level increased considerably when the hardening ratios increased from 0 to 1. Based on this observation, metallic auxetic metamaterials undergoing large deformation were dominated by the topology of the microstructures; this was independent of the properties of the base material.

## 5. Discussion and Conclusions

The findings of this study demonstrated a fundamental way to design a new class of 2D metallic auxetic metamaterials. The new design approach consisted of two important improvements compared with the previous approach for elastomer [10,11]. The first improvement was the inclusion of a geometric modification of the conventional structure to generate a new buckling-induced metamaterial. The second improvement was altering the initial geometry of a non-metallic auxetic metamaterial at the RVE level. The FE models were validated by experiments. A systematic parametric study was carried out using validated FE models on the effects of the plastic properties of the base material and the pattern scale factor.

It is interesting to note that a similar approach to that of the PSF has been also used by other researchers to direct the deformation path of soft metamaterials [40]. The effect of boundary conditions on the auxetic performance of our 2D auxetic metamaterial was also investigated. From the results of those investigations, the following conclusions can be drawn:

(1) The deformation process of the metallic auxetic metamaterial is influenced by boundary conditions; however, the auxetic behavior and its performance corresponding to different deformation processes are insensitive to boundary conditions.
(2) The auxetic behavior of our designed metallic metamaterial remains within the effective auxetic strain range.
(3) The effective auxetic strain can be controlled individually by PSF while maintaining a similarly negative value of Poisson’s ratio, especially when the PSF is in the range of 10% to 60%.
(4) The stiffness and strength of the metallic auxetic metamaterials can be individually controlled through adjustment of the properties of the base materials, while the negative value of Poisson’s ratio remains relatively constant.
(5) The loss of auxetic behavior in the metallic buckling-induced metamaterial is attributed to the localization of plastic collapse of RVEs.
(6) The results from FE simulation confirmed that the buckling-induced metamaterial would have auxetic behavior after the plastic hardening modulus is large than a certain value determined by the microstructures of metamaterials. These results confirm that the effectiveness of increasing plastic hardening ratio will restore the auxetic behavior of buckling-induced metamaterials.

**Figure 12:**
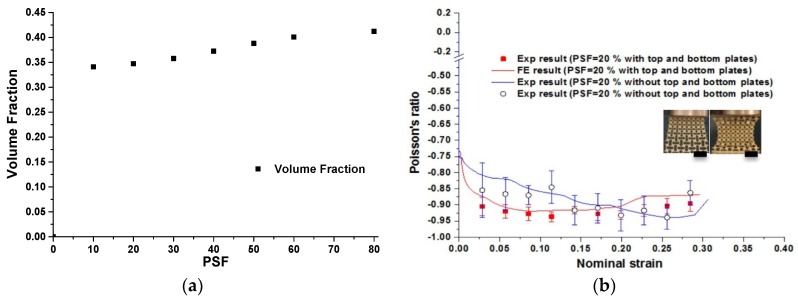
(**a**) Relation between PSF and corresponding volume fraction; (**b**) comparison of auxetic behaviors between buckling-induced design with PSF = 0%, buckling pattern-altered design without plates (Scale bar: 20 mm, load direction Y, strain rate: 5 × 10^−3^ S^−1^).

Except for those solid conclusions based on our numerical and experimental results, there are some concerns relating to the 2D microstructures of our metallic auxetic metamaterials. The mechanism of the resultant metallic auxetic metamaterials is the rotating of joints/squares. The analytical model of rotating interconnected squares was proposed independently and simultaneously by Ishibashi & Iwata [41] and Grima & Evans [39]. Thus, most of the auxetic behavior of the metallic auxetic metamaterial can be predicted by their model. To some extent, the PSF used to define the topology of the RVE can be equivalent to the rotation angle of the square units in the analytical model. The insensitive negative value of Poisson’s ratio of −0.9 with respect to PSF can be approximately explained by the negative value of Poisson’s ratio of −1 in a model of the rotating of joints/squares. However, there is a noticeable advantage to using PSF to design the microstructures of metallic auxetic metamaterials, besides tuneability. The volume fraction does not vary significantly through changing the PSF, as shown in Figure 12a. Thus, it can be revealed that a set of 2D auxetic metallic metamaterials that are obtained with different PSFs have similar volume fractions.

It should be noted that the Poisson’s ratio was measured in the center region of the specimens both in experiments and FE simulations. The nominal strain of the overall specimen was using to plot all strain-related curves. This method was used in all recent experimental-related research on auxetic structures and materials [10,11,23,29]. This measurement resulted in the insensitivity of the measured Poisson’s ratio with respect to strain localization. It will enlarge the negative strain range for auxetic behavior, evidenced by the constant negative Poisson’s ratio of −0.9 after its effective strain of 0.2 in Figure 6 and Figure 7. Based on the observed deformation of a metallic auxetic metamaterial that is presented in Figure 5c (*ε* = 0.2040), the walls of central rows of unit building cells are touching. However, the unit building cells of the top and bottom rows are not compacted completely. As mentioned before, the base martial of the specimen was made of ductile metal. Beyond the auxetic strain range, the metallic metamaterial can still be compressed further by plastic deformation of those not fully compacted layers. While the local strains in the central region remain nearly constant or are slightly compressed, the Poisson’s ratio will remain negative. Thus, the measurement of the Poisson’s ratio in the center region may not be representative of the whole specimen when the strain localization occurs, as shown in Figure 5b. We present those data just for comparison purposes; the accuracy of this measurement should be checked further. For a similar reason, we used the densification strain to define the end point for an effective strain range.

In order to study the influence of the boundary on the auxetic performance of a designed specimen, the top and bottom plates were removed so that the top and bottom surfaces of a new 2D designed metamaterial with PSF = 20% were not constrained. An experiment was conducted to reveal its effect on the deformation process. At the beginning of the test, a localized deformation occurred near the loading end. With further compression, a localized lateral inwards deformation developed from the top moving surface toward the bottom fixed surface. Interestingly, this deformation process led to obvious auxetic behavior. The auxetic *versus* strain curves of two specimens were put together and the results were presented in Figure 12b. Interestingly, the overall trend and the average values of this set of Poisson’s ratio curves were similar. These observations illustrated that although the deformation process of our metallic auxetic metamaterial under compression was significantly influenced by boundary conditions, the auxetic performance was insensitive to the boundary conditions.

It was revealed that the enhancement of the plastic-hardening ratio leads to restoring the auxetic behavior of buckling-induced auxetic metamaterials. According to Figure 9b, this effect is obvious when the hardening modulus is in the range of Ep/Es = 0 and Ep/Es = 0.3. Out of this range, the auxetic performance of models with different hardening ratios was similar and their differences could be neglected. This result is in accordance with the finding that the auxetic effect persists and becomes even stronger when the hardening modulus is in the range of h = 100 MPa and h = 1000 MPa by Dirrenberger *et al*. [25]. However, regarding the metallic auxetic metamaterials with altered microstructures, the influence of an enhancement of the plastic-hardening ratio on auxetic performance is negligible.
